# Integration of the Forced-Choice Questionnaire and the Likert Scale: A Simulation Study

**DOI:** 10.3389/fpsyg.2017.00806

**Published:** 2017-05-18

**Authors:** Yue Xiao, Hongyun Liu, Hui Li

**Affiliations:** ^1^School of Psychology, Beijing Normal UniversityBeijing, China; ^2^Beijing Key Laboratory of Applied Experimental Psychology, School of Psychology, Beijing Normal UniversityBeijing, China

**Keywords:** mixed test format, forced-choice questionnaire, Likert scale, personality test, simulation

## Abstract

The Thurstonian item response theory (IRT) model allows estimating the latent trait scores of respondents directly through their responses in forced-choice questionnaires. It solves a part of problems brought by the traditional scoring methods of this kind of questionnaires. However, the forced-choice designs may still have their own limitations: The model may encounter underidentification and non-convergence and the test may show low test reliability in simple test designs (e.g., test designs with only a small number of traits measured or short length). To overcome these weaknesses, the present study applied the Thurstonian IRT model and the Graded Response Model to a different test format that comprises both forced-choice blocks and Likert-type items. And the Likert items should have low social desirability. A Monte Carlo simulation study is used to investigate how the mixed response format performs under various conditions. Four factors are considered: the number of traits, test length, the percentage of Likert items, and the proportion of pairs composed of items keyed in opposite directions. Results reveal that the mixed response format can be superior to the forced-choice format, especially in simple designs where the latter performs poorly. Besides the number of Likert items needed is small. One point to note is that researchers need to choose Likert items cautiously as Likert items may bring other response biases to the test. Discussion and suggestions are given to construct personality tests that can resist faking as much as possible and have acceptable reliability.

## Introduction

Personality tests are widely used in personnel selection situations, yet the authenticity and validity of the results are controversial. Conventional personality tests, which often use multidimensional Likert-type scales, may lead to many kinds of response biases, such as the halo effect and impression management (Morrison and Bies, 1991; Cheung and Chan, 2002). When these scales were used in personnel selection, respondents can easily fake their replies to increase their chances of being employed, which undermines the validity of personality tests and hiring decisions (Mueller-Hanson et al., 2003; Komar et al., 2008; Goffin and Boyd, 2009; Honkaniemi et al., 2011).

Many studies have indicated that the forced-choice personality questionnaires can resist faking effectively (e.g., Jackson, 2000; Bowen et al., 2002; Cheung and Chan, 2002; Christiansen et al., 2005; Hogan, 2005; Bartram, 2007). However, the traditional scoring method of this type of questionnaires produces ipsative data, which poses some analytical challenges (e.g., Dunnette et al., 1962; Tenopyr, 1988; Greer and Dunlap, 1997; Loo, 1999; Bowen et al., 2002; Meade, 2004). For example, factor analysis and variance analysis cannot be used and more important, the test reliability is low. But the effect of ipsative data could be weakened when the test consists of a fairly large number of subtests and the correlations between them are medium (Bartram, 1996). Unfortunately, these conditions are difficult to achieve in practice.

To resolve the problems of ipsative data fundamentally, psychometric scientists have constructed some item response theory (IRT) models in recent years. One of them is the Thurstonian IRT model of Brown (2010) and Brown and Maydeu-Olivares (2011), which has fairly large influence and is relatively simple to use. The model can estimate the true score of the respondent directly through his/her response pattern. Thus, researchers can analyze the normative scores and estimate the test reliability using the test information function.

Nevertheless, the forced-choice designs may still have their own limitation and the Thurstonian IRT model can only solve part of problems of ipsative data. An accurate recovery of trait scores and parameters based on the model largely depends on the test design, especially on the number of constructs measured (Brown and Maydeu-Olivares, 2011). If the test measures a small number of constructs, there exists estimation problems and test reliability is quite low for some conditions. Moreover, the model identification is an issue of concern in practice, which may be encountered in some test designs. Some reasons for underidentification are not easy to find out. And the current solution for identification under some conditions is to constrain some parameters to their true values that are often unknown in practice.

To improve the poor performance of the Thurstonian IRT model in certain conditions and to further address the problems of ipsative data, the present study uses the combination of the Thurstonian IRT model and the Graded Response Model for a type of different test formats that consists of the forced-choice blocks and the Likert-type items. And the Likert items should have low social desirability for the resistance of the test to faking. The aim of this study is to investigate how the mixed response format performs under various conditions, compare the performance of the mixed format and the forced-choice format and provide evidence and guidance for designing personality questionnaires that can avoid faking. In this article, we refer to “traits,” but the same method also applies to questionnaires measuring other types of psychological constructs, such as motivation and attitudes. And we assume that items are from the same dimension if they measure the same construct or trait.

The article is structured into five sections. In the first section, we provide a review of ipsative data and the Thurstonian IRT model. The second section is a brief mathematical introduction of the combined model. The Monte Carlo simulation method and results are presented in the third and the fourth section, respectively, to illustrate the properties and the performance of the mixed response format. In the fifth section, we summarize the research findings, discuss their implications and the limitations and provide guidance about personality test design.

## Literature review

The forced-choice form at test, a test-constructing technique, presents two or more statements in a comparative fashion. The items within each block are paired in terms of an index of preference and discrimination (e.g., social desirability). For blocks of only two items, respondents are asked to choose the one that best describes them. When there are more than two statements in a block, respondents are required to rank the items or select two items that are “most like me” and “least like me,” respectively. In a forced-choice test in which one block is composed of two matched statements, if the traditional scoring method is used, the score of a respondent in a dimension equals the number of statements he/she chooses measuring that dimension. The scoring method is similar when each block consists of more than two items. Thus, for any individual, the sum of his/her scores is a fixed value, which generates *ipsative* data.

A number of studies have indicated that ipsative data make trouble for the explanation and analysis of the scores for the relative nature of scores (Hicks, 1970; Tenopyr, 1988; Dunlap and Cornwell, 1994; Meade, 2004). In addition, the data distort construct validity and criterion-related validity of the test (Brown and Maydeu-Olivares, 2013).

More important, the ipsative scoring distorts reliability estimates (Meade, 2004; Brown and Maydeu-Olivares, 2013). The ipsative data violate the hypotheses of the classical test theory (CTT) because of the comparative nature of the data, while the calculation formulas in the traditional estimation method of reliability are all based on CTT. Accordingly, the traditional estimation method of test reliability is inappropriate for the forced-choice tests. Baron (1996) pointed out that the use of Cronbach's alpha would underestimate the reliability of the forced-choice test. As the classical solution, increasing the number of subscales can alleviate the impact of ipsativity to some extent. Several researchers have showed that the negative effect of ipsative data could be weakened when the measuring instrument was composed of 30 or more subtests (e.g., Baron, 1996; Bartram, 1996). The “subscales” and “subtests” mentioned here and later both refer to dimensions in the instrument. The items that measure the same trait belong to the same subscale or the same subtest. However, many traits measured in the test still do not solve the problems of ipsativity and are fairly difficult to achieve in practice (Brown and Maydeu-Olivares, 2012, 2013).

As a solution to the problems brought by ipsative data, Brown and Maydeu-Olivares (2011) proposed the Thurstonian IRT model by embedding latent traits within Thurstone's (1927, 1931) Law of Judgement. The model is estimated by a structural equation model (SEM). Brown and Maydeu-Olivares (2013) showed the higher test reliability and validity of the forced-choice test scoring with the model than the traditional method, using the Customer Contact Styles Questionnaire (CCSQ). Moreover, using the Occupational Personality Questionnaire 32, Joubert et al. (2015) found that the IRT-scored forced-choice test and the Likert-type personality test could yield similar results, such as similar test reliability.

In spite of these results, the studies have one thing in common: their measurement instruments are also composed of a number of subtests, for example, 16 dimensions in the CCSQ and 32 subscales in the Occupational Personality Questionnaire 32. A stable and accurate estimation of the Thurstonian IRT model entails some requirements specific to the forced-choice format. Brown and Maydeu-Olivares (2011) discussed factors that might affect performance of the Thurstonian IRT model through simulations and an empirical study. More concretely, there are three points. First, nearly half of the forced-choice binary outcomes are from a comparison of items keyed in opposite directions. When this is met, the IRT model can get a good estimation even if the number of traits is small or the correlations between traits are strongly positive. Second, if the number of traits is large and the intertrait correlations are not strongly positive, the trait and parameter recovery can be good, even when there are only positively keyed items. Third, a large number of statements measuring each trait is beneficial to model estimation.

The recommendations of Brown and Maydeu-Olivares (2011) are for the general forced-choice tests. But some special problems might occur in the forced-choice tests with resistance to faking. For personality tests, there usually exists a clear difference in social desirability between the two items keyed in opposite directions in a pair. This difference could easily trigger faking responses. People always tend to choose the positive-worded items in pairs that consist of items keyed in opposite directions. As Brown and Maydeu-Olivares (2011) also pointed out, the accuracy of latent trait and item parameter recovery largely depended on the number of traits measured when each block only consisted of items keyed in the same direction. Therefore, the number of traits in forced-choice designs plays an important role in the estimation of the Thurstonian IRT model.

The force-choice test format may also pose a problem to the identification of the Thurstonian IRT model. For most forced-choice designs, to identify a Thurstonian IRT model, it generally suffices to set all variances of latent traits and the uniqueness of one item per block to 1. But when the test measures only two traits with blocks of two items, the factor loading of the first item in each trait should be set to its true values (Brown and Maydeu-Olivares, 2012). When factor loadings of two items within the same block are equal or indistinguishable, two loadings within each block may need to be constrained to be equal and one correlation between the latent traits should be set to its expected value (Brown and Maydeu-Olivares, 2012). However, researchers always do not know the true values of factor loadings or intertrait correlations in practice. This may cause the constraints that researchers make to be inconsistent with the theory or the fact, and then decrease the goodness of model fit and the accuracy of estimation. Second, it may not be easy to discover some types of empirical underidentification. For example, it is not easy to judge whether the factor loadings of item pair. The model users are required to have relevant knowledge to deal with these cases.

The main cause of the problems listed above is that the forced-choice designs produce binary outcomes from the comparison of items indicative of different traits. When only positively items are employed, the differences between the traits but not their sums are recovered, providing insufficient information for the absolute locations of traits. Therefore, trait recovery and item parameter recovery are both poor. This problem is particularly prominent when the test measures a small number of traits. In contrast, Likert-type items are a form of rating only one statement at a time, providing complete information about the absolute location of the trait that the item measures. This type of items does not pose the above problems of the forced-choice designs.

Considering the characteristics of these two response formats, we consider a test format that contains the two type of items and we use the combination of the Thurstonian IRT model and the Graded Response Model for this test format. A similar idea of combining forced-choice questionnaires and Likert scales was quite popular back in the day when forced-choice questionnaires resulted in ipsative scale scores. The examples of tests using this exact format are Inventory of Management Competencies (IMC) and the CCSQ. In these tests, each item has to be rated on a five-point Likert scale, while all statements are further grouped in quads and the rater also are required to indicate which of each set of four items is most true and least true of the target subject. The scale scores were derived by summing the ipsative item scores and the normative item scores. In this article, we use different statements as Likert items and forced-choice items, which shortens the test length, and the Likert items should have low social desirability for the resistance of the test to faking. We also use IRT models for scoring and estimation instead. More important, both IMC and CCSQ still have 16 dimensions but we investigate the performance of this combined format when a fewer number of traits are measured.

About the model, we use the combination of the Thurstonian IRT model and the Graded Response Model (GRM; Samejima, 1969). One of the advantages of IRT is providing a flexible mechanism for the use of indicators with different number of categories. And the idea of combining indicators of different types to infer measurement of latent traits in SEMs is popular and has been used widely (e.g., in aptitude testing). Therefore, we apply the two IRT models for estimation and linking the indicators to the latent traits. In addition, we hypothesize that Likert items can provide information of the scale of latent traits. Accordingly, in our simulations, no constraints are set to any factor loadings in test designs measuring two traits with Likert items.

One possible object is that adding Likert items may influence the resistance of the forced-choice questionnaire to many other response biases. From this perspective, Likert items should be as few as possible. Therefore, we should know how the proportion of Likert items in the test would influence model estimation and whether Likert items are needed in practice. But previous research rarely discusses these problems. We investigate the influence of the proportion of Likert items in the present study. Given the characteristics of two response formats, we conjecture that a few Likert items might suffice to help solve the estimation problems of the Thurstonian IRT model in certain conditions. If the conjecture is right, the negative impact of Likert items on resistance to response biases could be low. Researchers can also use other approaches in choosing Likert items to further alleviate the negative impact of response biases as much as possible.

To summarize, we expect that the combined model can solve the problems of the Thurstonian model and provide a precise estimation of trait scores and item parameters, even when the number of traits is fairly small (e.g., two traits).

## The combined model

**Figure 1:**
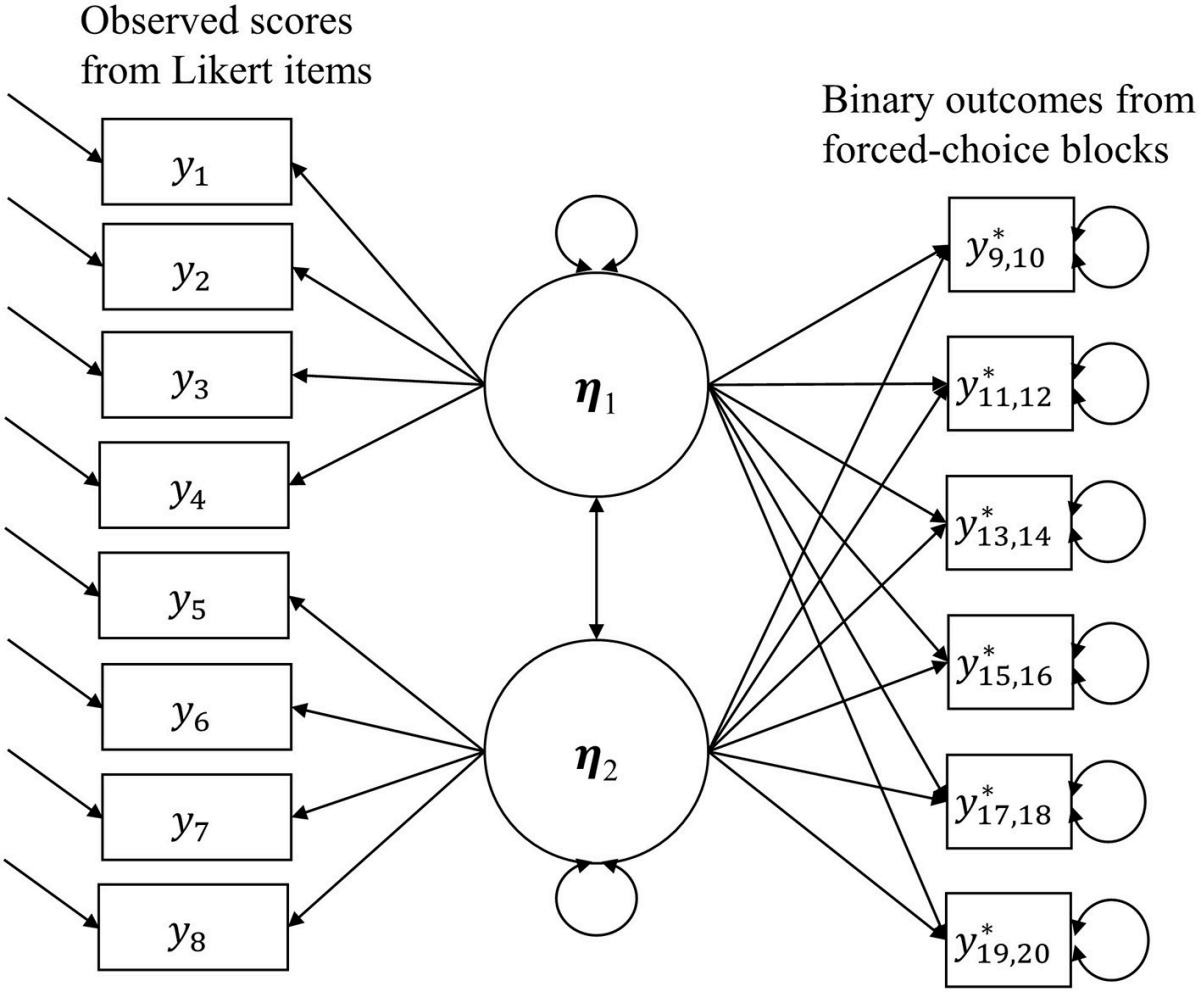
**The combination of the Thurstonian IRT model and the Graded Response Model**.

The combined model is in Figure 1, the right half of which is still the Thurstonian IRT model, but the model uses the observed scores given by Likert items at the same time when estimating latent traits. And the estimation for Likert items is the same as that for the graded response model.

Therefore, the total information on trait η_*a*_ given by the test is the sum of the information provided by all binary outcomes and Likert items contributing to the measurement of trait η_*a*_, that is,

(1) $$I_{T}^{a}\left(\eta \right)=I^{a}\left(\eta \right)+I_{L}^{a}\left(\eta \right).$$

In the Equation (1), *I*^*a*^(η) is the total information provided by all binary outcomes contributing to the measurement of trait η_*a*_. $I_{L}^{a}\left(\eta \right)$ is the information provided by all Likert items measuring trait η_*a*_.

The *SE* of the estimated score ${\hat{\eta }}_{a}$ can be calculated easily and the empirical reliability can then be obtained, as can be seen in Equations (2) and (3), respectively.

(2) $$SE\left({\hat{\eta }}_{a}\right)=\frac{1}{\sqrt{I_{T}^{a}\left(\hat{\eta }\right)}}$$

(3) $$\rho =\frac{\sigma_{p}^{2}-{\bar{\sigma }}_{error}^{2}}{\sigma_{p}^{2}}$$

## Methods

### Design

A Monte Carlo simulation study was performed and the forced-choice blocks presented are presented in the form of item pairs in which the two items measure different traits.

Thirty-two conditions were examined in this simulation study by crossing the following four factors: (a) the number of traits (2 or 5); (b) test length, expressed as the ratio of the number of all questions in the test to the number of traits (5:1 or 10:1; for example, 5:1 means that the test comprised 10 questions when there were two traits and 25 questions when there were five traits); (c) the percentage of Likert items (0, 20, 40, or 60; for example, 20% Likert items in the design with two traits and 10 questions indicates two Likert items and eight forced-choice blocks in the questionnaire); (d) the proportion of pairs composed of items keyed in opposite directions (0 or 20%), which means the ratio of the number of pairs in which comparisons are between opposite directions to the total number of Likert items and the blocks. It should be noted that all the Likert items were positively worded and there were equal numbers of Likert items per trait. Under all conditions, the latent traits were all normally distributed, the correlation between the traits was set to 0.2, and the proportion of negative statements was about 30% of all statements.

In test designs measuring two traits with Likert items, no factor loadings needs constraints when the model is analyzed. But in pure forced-choice designs, the designs with 0% of Likert items, measuring two traits, the factor loading of the first item in each trait still needs to be set to its true value for model identification.

### Data generation

The true item parameters were drawn from a uniform distribution: between −0.8 and 0.8 for intercepts (μ) and between 0.45 and 0.9 for absolute values of factor loadings (λ).

Then, according to the test design conditions, items from different traits were matched yielding forced-choice pairs while a certain percentage of Likert items were retained to construct 32 versions of tests.

Finally, the responses from subjects were generated corresponding to the different test designs. The latent scores were subject to a standard normal distribution and true uniquenesses were all fixed at one. Specifically, use Mplus to generate latent trait scores (η) and errors (e) for each subject. According to the Thurstonian IRT model of Brown and Maydeu-Olivares (2011), each item to be ranked would elicit a utility. We use *t*_*i*_ as the latent utility associated with Item *i*. For a forced-choice block, the difference of the latent utilities between two paired items could be computed for each subject. The differences were then transformed into a dichotomous variable y_1_ according to whether the difference values are <0. If the difference is <0, y_1_ is equal to 0. If not, y_1_ is 1. The variable y_1_ represents the responses to the forced-choice block. For a Likert-type item, the distribution of utilities for each item from all participants could be approximated to a normal distribution. According to the probabilities of the distribution, the latent utilities of each item could be transformed into a rating scale from 1 to 5. Then, the Likert rating scores can be obtained.

Using R and Mplus, a total of 100 replications were obtained for each condition and the sample size was 2,000 observations for all conditions.

### Model evaluation

To compare the performance of the combined model under different conditions, three aspects of the model were investigated, that is, model convergence, the accuracy of parameter estimation, and the precision of latent trait recovery.

#### Model convergence

In the simulation study of Brown and Maydeu-Olivares (2011), the convergence rate was usually unable to reach 100% in simple designs, that is, there always existed some replications that failed to be identified and estimated. However, a higher model convergence rate indicates that the model is more stable in the corresponding condition. Thus, the present study expected to investigate the performance of the mixed response format from the aspect of model convergence rate. Note that the convergence rate is the proportion of replications that successfully converged in 100 replications under each condition.

#### Item parameter recovery

For all conditions investigated, the root-mean-square error (RMSE) was used to assess the accuracy of the estimation of parameters (including thresholds of forced-choice items, factor loadings of Likert items, factor loadings of forced-choice items, and intertrait correlations) and their *SE*s. The index illustrates the deviation of the estimated value of the parameter from its true value, and a smaller RMSE shows that the item parameter recovery is more accurate. This can be calculated by Equation (4).

(4) $$RMSE({\hat{f}}_{p})=\sqrt{\frac{1}{R}\sum_{r\;=\;1}^{R}{\left({\hat{f}}_{pr}-f_{p}\right)}^{2}}$$

#### Latent trait score recovery

To evaluate the trait recovery, actual reliability described in Brown and Maydeu-Olivares (2011) was used. Estimated scores for each latent trait can be obtained using Mplus and these are correlated with the true trait scores. The square of this correlation is actual reliability and its value ranges from 0 to 1, where a larger value indicates that the true score recovery is more accurate and test reliability is higher.

## Results

### Model convergence

Table 1 provides the model convergence rates under all conditions investigated. Compared with the designs without Likert items, the inclusion of some Likert items helps the model to converge successfully, especially under conditions which are not conducive to its estimation, such as the designs with a small number of traits, short length, or no pairs composed of items keyed in opposite directions. With regard to the designs with a relatively large number of traits (five traits) and a large proportion (40 and 60%) of Likert items, the model estimation proceeded successfully for all 100 replications.

**Table 1:** **Model convergence rates (%) under all conditions**.

**Number of traits**	**Test length^a^**	**Proportion of pairs composed of items keyed in opposite directions (%)**	**Percentage of Likert items**			
			**0%**	**20%**	**40%**	**60%**
2	5:1	0	83	94	100	100
		20	95	100	100	100
	10:1	0	98	100	100	100
		20	100	100	100	100
5	5:1	0	100	100	100	100
		20	100	100	100	100
	10:1	0	100	100	100	100
		20	100	100	100	100
Average			97	99	100	100

a *The test length is expressed as the ratio of the number of all questions in the test to the number of traits*.

It should be particularly specified that, in addition to the low convergence rates, there were also several extreme estimated *SE*s under the four conditions with two traits measured and no Likert items. A test without Likert items means that it is purely a forced-choice questionnaire and analyzed by the Thurstonian IRT model. However, the similar things occurred when the test included only 20% of Likert items with two traits measured and no pairs composed of items keyed in opposite directions and short length. Table 2 presents these five conditions and the numbers of replications in which some of the estimated *SE*s were larger than 10 under these conditions.

**Table 2:** **Conditions with extreme estimated SEs and the corresponding numbers of replications in which some estimated SEs were larger than 10**.

**Test length^a^**	**Proportion of pairs composed of items keyed in opposite directions (%)**	**Percentage of Likert items (%)**	**The number of replications with the following cases**		
			**At least one forced-choice threshold's *SE* > 10**	**At least one forced-choice loading's *SE* > 10**	**At least one Likert item loading's *SE* > 10**
5:1	0	0	10	13	0
	0	20	0	0	8
	20	0	5	8	0
10:1	0	0	3	4	0
	20	0	2	2	0

*The five designs listed all measure two traits*.

a *The test length is expressed as the ratio of the number of all questions in the test to the number of traits*.

To avoid the effect of those estimated results that were unacceptable on the following analysis, the present study selected those replications that converged successfully and for which all estimated *SE*s were smaller than 10, for the subsequent analysis.

### Item parameter recovery

**Figure 2:**
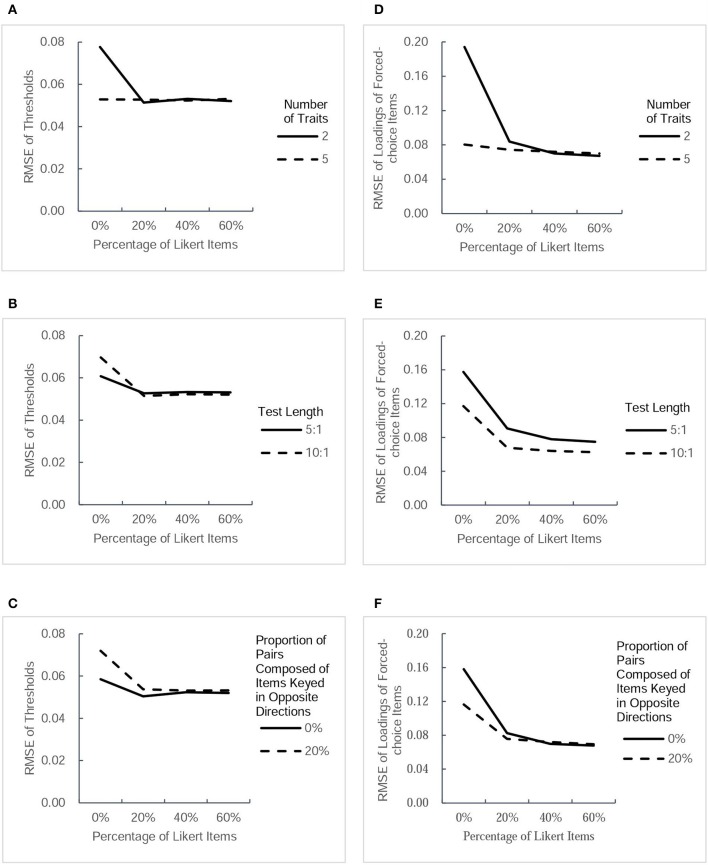
**RMSE-values for two type of parameter estimates (thresholds and factor loadings of forced-choice items) under different conditions**. Each column presents the change tendency of one type of parameter estimates as the percentage of Likert items increases in different conditions. The panels **(A–C)** correspond to the RMSE of thresholds as a function of percentage of Likert items in different conditions of three factors. The panels **(D–F)** correspond to the RMSE of forced-choice items' loadings as a function of percentage of Likert items in different conditions of three factors.

**Figure 3:**
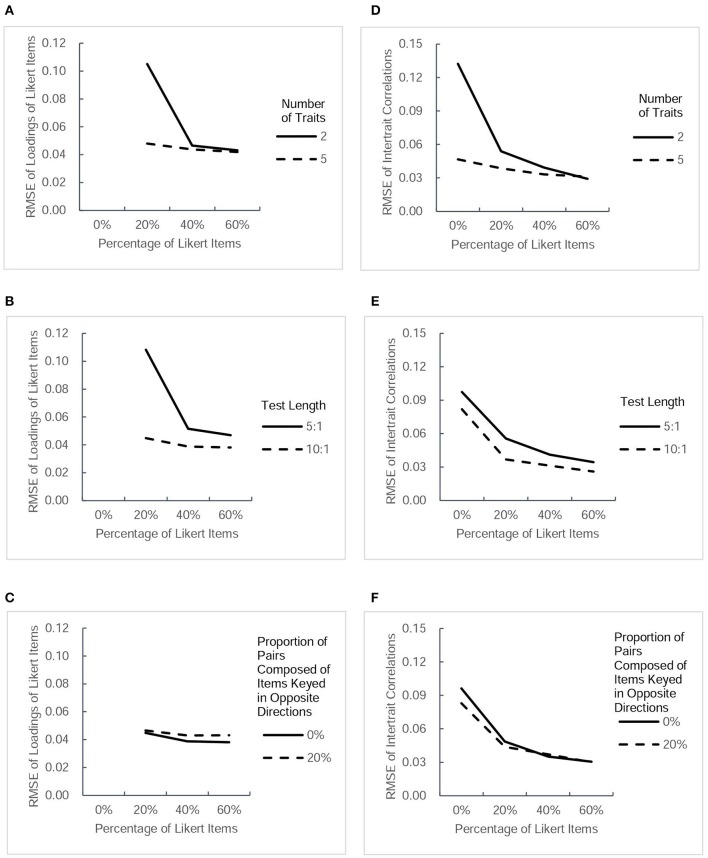
**RMSE-values for another two types of parameter estimates (factor loadings of Likert items and intertrait correlations) under different conditions**. Each column presents the change tendency of one type of parameter estimates as the percentage of Likert items increases in different conditions. The panels **(A–C)** correspond to the RMSE of Likert items' loadings as a function of percentage of Likert items in different conditions of three factors. The panels **(D–F)** correspond to the RMSE of intertrait correlations as a function of percentage of Likert items in different conditions of three factors.

**Figure 4:**
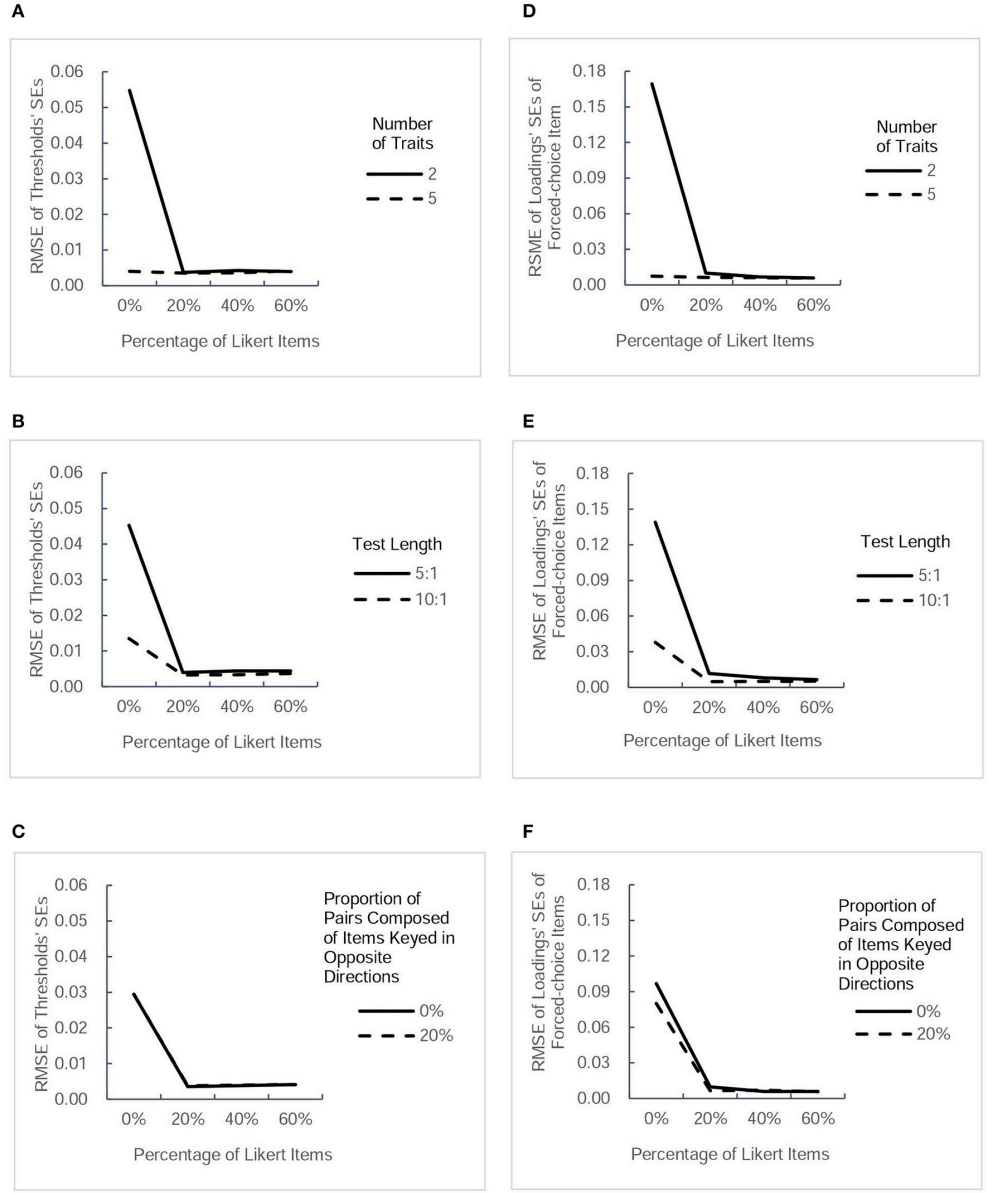
**RMSE-values for estimated standard errors (*SEs*) of two type of parameter estimates (thresholds and factor loadings of forced-choice items)**. Each column presents the change tendency of one type of estimated *SEs* as the percentage of Likert items increases in different conditions.The panels **(A–C)** correspond to the RMSE for thresholds' *SEs* as a function of percentage of Likert items in different conditions of three factors. The panels **(D–F)** correspond to the RMSE for *SEs* of forced-choice items' loadings as a function of percentage of Likert items in different conditions of three factors.

**Figure 5:**
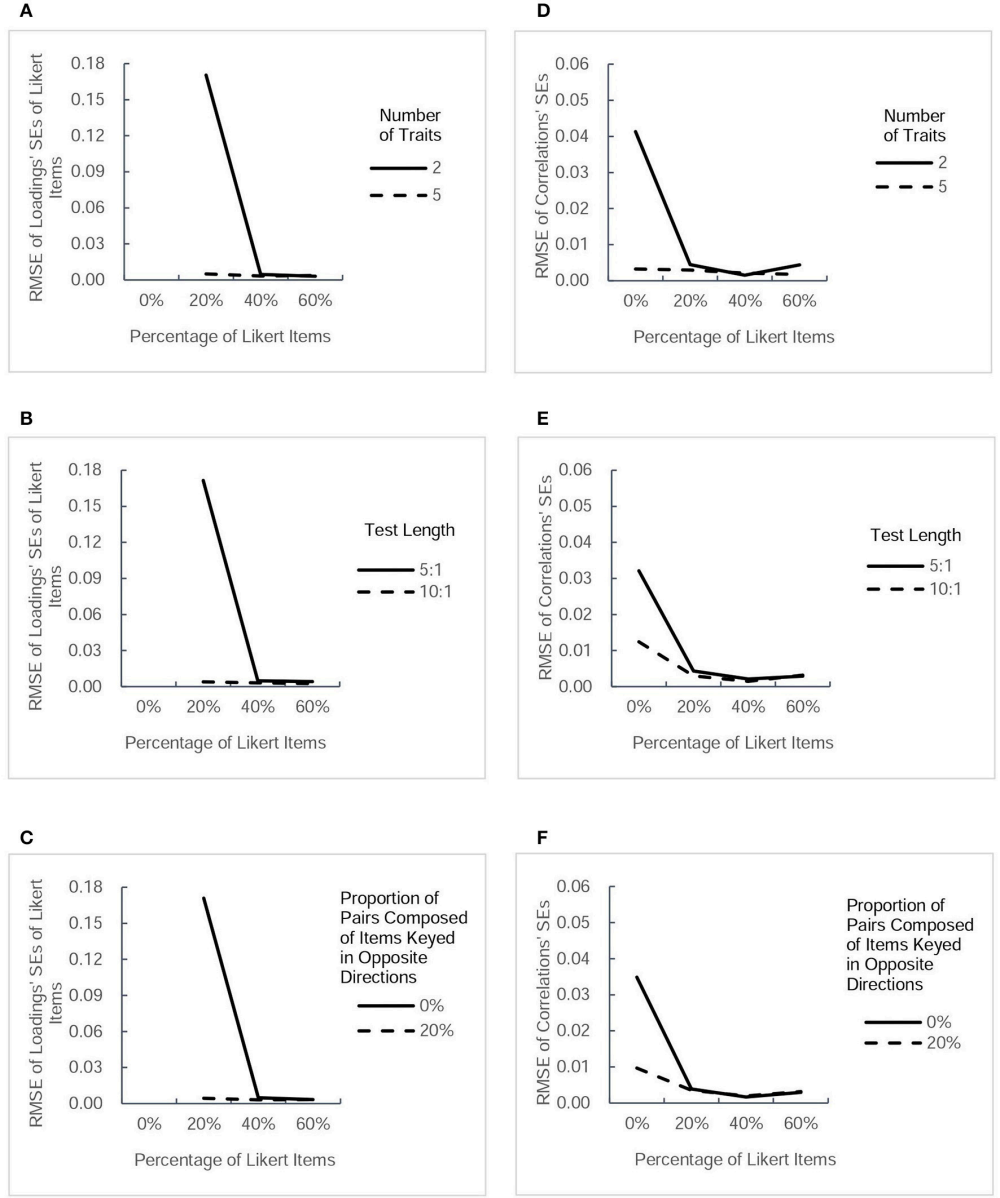
**RMSE-values for estimated standard errors (*SEs*) of another two types of parameter estimates (factor loadings of Likert items and intertrait correlations)**. Each column presents the change tendency of one type of estimated *SEs* as the percentage of Likert items increases in different conditions.The panels **(A–C)** correspond to the RMSE for *SEs* of Likert items' loadings as a function of percentage of Likert items in different conditions of three factors. The panels **(D–F)** correspond to the RMSE for intertrait correlations' *SEs* as a function of percentage of Likert items in different conditions of three factors.

The accuracy of parameter estimation was evaluated for both parameter estimates and *SE* estimates. For the 32 conditions, the table in the Supplementary Material lists the values of RMSE of four types of parameter estimate and those of their estimated *SE*s. For a more intuitive description, Figures 2, 3 present RMSE-values of estimated parameters under different conditions, and Figures 4, 5 present RMSE-values of estimated *SE*s corresponding to each type of parameters.

#### RMSE of parameter estimates

Figure 2 depicts the values of RMSE across the estimated thresholds and factor loadings of forced-choice items. Figure 3 depicts the RMSE-values of factor loadings of Likert items and intertrait correlations. Because a smaller RMSE-value represents greater precision of the estimation, it can be seen that the inclusion of Likert items improved the estimation accuracy of the thresholds and factor loadings of forced-choice item and intertrait correlations somewhat, regardless of other factors. The improvement in the precision of estimation was found to be more obvious when the test measured fewer traits, the test was shorter, or there were no pairs composed of items keyed in opposite directions. However, there seemed to be only a small difference in the accuracy of parameter estimates among designs with 20, 40, and 60% of Likert items under all conditions, except for the Likert item factor loadings. For this parameter, under the designs with two traits measured, short length, or no pairs composed of items keyed in opposite directions, the RMSE-values of its estimates decreased when the percentage of Likert items increased from 20 to 40%, but remained almost the same when the percentage of Likert items was changed from 40 to 60%. However, the precision of estimation showed little change as the percentage of Likert items increased under the designs with five traits, long length, or including pairs composed of items keyed in opposite directions. In general, after Likert items were added into the test, increasing their proportion had little effect on improving the precision of most parameter estimates, regardless of other influential factors.

#### RMSE of estimated SEs of parameters

Figure 4 presents the values of RMSE across the estimated *SE*s of thresholds and factor loadings of forced-choice items, and Figure 5 describes the RMSE-values of estimated *SE*s of factor loadings of Likert items and intertrait correlations under different conditions. Generally, the trends in the precision of estimated *SE*s were very similar to the trends of estimated parameters in Figures 2, 3. Combining the Likert test with the forced-choice test could substantially improve the accuracy of estimated *SE*s of thresholds and factor loadings of forced-choice items and intertrait correlations, especially in the designs with fewer traits, shorter length, or no pairs composed of items keyed in opposite directions. It also appeared that the accuracy of most estimated *SE*s changed little among designs with different percentage of Likert items, except the factor loadings' *SE*s for both types of items. With regard to the estimation accuracy of loadings' *SE*s of Likert items, its variation trend was exactly the same as that of Likert item loading estimates. The only difference between the tendency of loadings' *SE*s of forced-choice items and that of forced-choice item loadings estimates was the substantial decrease in its RMSE-values when the percentage of Likert items was increased from 20 to 40%. However, taken as a whole, 20% of Likert items also seemed sufficient to improve the estimation precision of the *SE*s of most parameters.

### Latent trait score recovery

**Figure 6:**
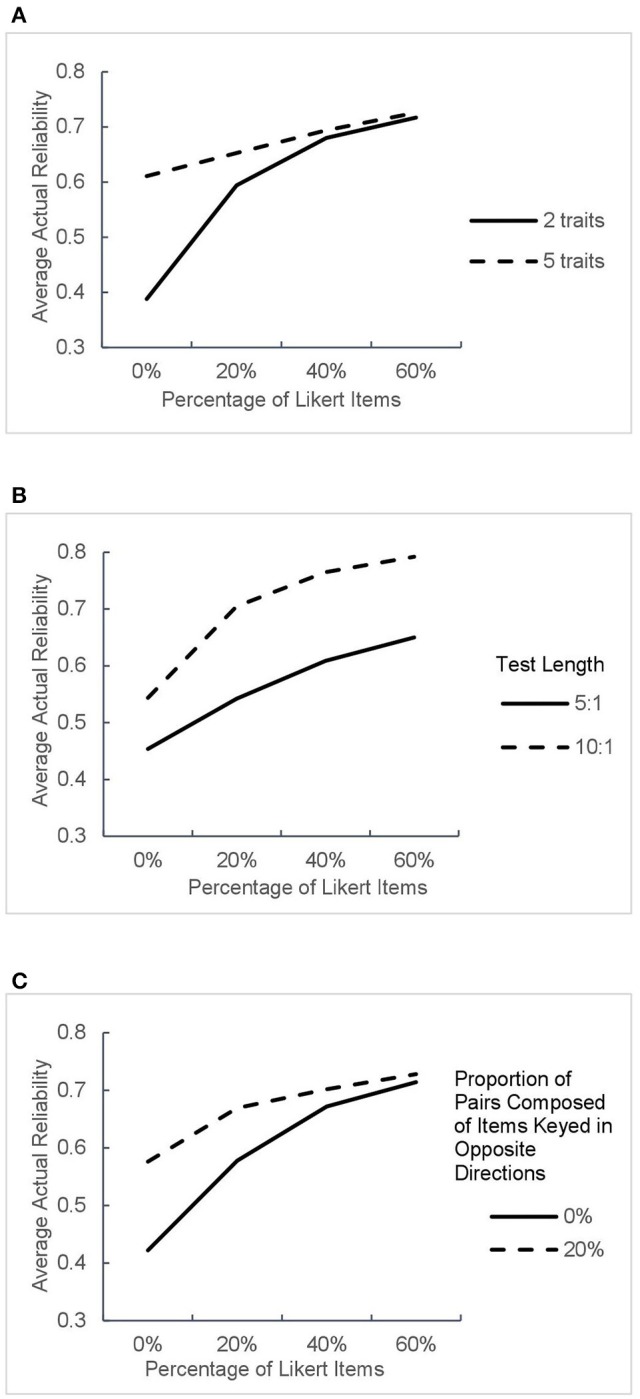
**Average actual reliabilities in different test design conditions**. The panels **(A–C)** correspond to average test reliability as a function of percentage of Likert items in different conditions of three factors.

The average actual reliabilities estimated under all 32 conditions are shown in Table 3 and Figure 6.

**Table 3:** **Average actual reliabilities under all conditions**.

**Number of traits**	**Test length^a^**	**Proportion of pairs composed of items keyed in opposite directions (%)**	**Percentage of Likert items**			
			**0%**	**20%**	**40%**	**60%**
2	5:1	0	0.238	0.477	0.586	0.619
		20	0.489	0.568	0.607	0.662
	10:1	0	0.266	0.616	0.746	0.797
		20	0.556	0.753	0.797	0.807
5	5:1	0	0.550	0.559	0.611	0.659
		20	0.539	0.599	0.644	0.676
	10:1	0	0.634	0.699	0.760	0.799
		20	0.720	0.760	0.771	0.786
Average			0.499	0.629	0.690	0.726

a *The test length is expressed as the ratio of the number of all questions in the test to the number of traits*.

As can be seen from Figures 6A–C, the inclusion of Likert items in the test obviously improved the actual reliability and the increase was greater under the designs with two traits or no pairs composed of items keyed in opposite directions. When the test contained Likert items, the growth in reliability induced by the increased proportion of Likert items slackened under all conditions, which is consistent with the results of parameter recovery in the previous section.

## Discussion

The present study integrates the Thurstonian IRT model and the Graded Response Model for a test format which combines the forced-choice questionnaire with the Likert scale. We found that the forced-choice format performed poorly under certain conditions, which is consistent with previous research findings, while the mixed response format was better, especially when the number of traits measured was small.

Does the mixed response format help to solve the problems of forced-choice designs? From the simulation, we found a remarkable improvement in the accuracy of parameter and true score recovery of the combined model, compared with those of the Thurstonian IRT model. In addition, the average convergence rate of the combined model was demonstrably greater than that of the Thurstonian IRT model and the IRT model obtained some extreme estimated standard errors in some designs measuring two traits. These indicate the model estimation of the mixed test format is more stable. Most important, the difference between the two models is remarkable under the simple test designs (designs measuring two traits, with only pairs using positively-worded items, or a small number of items), where the performance of the Thurstonian IRT model is unacceptable. But when the test measures more traits, has more items or includes pairs composing of opposite keyed items, the performance of the two models become similar. Further, the model may need less constraints when Likert items are added into the test.

The findings prove that Likert items provide the model estimation with the complete information on the absolute latent trait scores. Accordingly, the latent trait scale can be easily defined and the information is sufficient for the model to produce a stable and accurate estimation, particularly when the test measures only a small number of traits. From this view, the combined model only needs fewer constraints than the IRT model for model identification. In conclusion, the mixed test format does provide a solution for low reliability and model identification problem of the forced-choice format under certain conditions.

One possible object to the conclusion is that we eliminated replications whose estimated standard errors were larger than 10 to avoid the influence of extreme values. Choosing 10 as the cut-off point is out of the following considerations. If we use the relative criterion, such as the standard score of 3 or greater as a cut-off point, we should screen each type of standard errors. But extreme values usually occur only in standard errors of some parameters. Then we may delete more replications than we should, making the estimation results seem precise but actually are not. Thus, the absolute criterion seems more appropriate. However, there is no generally accepted absolute criterion to judge whether the estimated standard errors were in a normal range. Accordingly, we counted the number of replications which had at least one estimated standard error was larger than 1, 5, 10, 20, or 50, respectively. We found that using 10 as a cut-off point, we would not delete too many replications and could get the right tendency of the results, as seen in the section of results. But no matter what, obtaining extreme estimated standard errors has already proved the unstable model estimation of the forced-choice designs.

How many Likert items does the test need? The simulation results indicated that 20% of Likert items in the test almost provide sufficient information for stable and accurate estimation in the conditions where the Thurstonian IRT model performed poorly. Among these conditions, the most complex one is that the test measures two traits and has a total of 20 questions (the ratio of the number of questions to the number of traits is 10:1). In this test, 20% of Likert items equals only four Likert items and each trait has two of them. Besides, when the number of traits measured is large or the test length is relatively long, the forced-choice questionnaire can perform well and there is no need to add Likert items. Hence, the number of these items needed in certain designs is not large.

The most obvious limitation of this research is about the resistance to response biases of the mixed test formats. Although the number of Likert items needed is small, they may bring different types of response biases to the test. The present study is to explore an available solution for problems which forced-choice design may encounter in application, which is from the aspect of statistical models and model estimation. Things are more complex than this in practice. Researchers should choose Likert items with great cautions. To reduce the probability of faking and social desirability responding, we emphasize that items with low social desirability should be considered as Likert items. The items may be those simple and objective questions (Skinner, 1978). There have been some approaches control some other response biases taken from Likert items. For example, non-judgmental or non-threatening items can be used as Likert items if the test is about sensitive issues (Johnson, 1970). Use of balanced Likert scales or just logically opposite items can help avoid the acquiescent response bias (Winkler et al., 1982; Ray, 1990). Later, because of statistical problems taken by this practice, bidirectional response options are proposed to be an alternative (Barnette, 2000). Therefore, if researchers can pick appropriate Likert items, the test can still possess good resistance to response biases. But if not, the test may encounter additional problems. Moreover, how to select good Likert items is always a complex issue worthy of investigation.

The study also has some other limitations. The forced-choice tests have various formats because the forced-choice blocks can have different sizes. The mixed response format as presented here uses only the simplest forced-choice format, blocks composed of two items. Besides, the factor loadings of Likert items in our simulation were drawn from a uniform distribution ranging from 0.45 to 0.95. Accordingly, the conclusion of the current study cannot be generalized to the conditions where the Likert items have low factor loadings. It is quite possible that the results may be different if each forced-choice block has more than two items or the factor loadings of Likert items are smaller. Researchers interested in this test format and scoring method could investigate how the mixed response format performs in these conditions. A final point to note is that the present study is a simulation study to compare model performance among different conditions. Future study is also required to examine the results in empirical examples.

Furthermore, there exist other IRT models for scoring forced-choice tests, such as the multi-unidimensional pairwise-preference (MUPP) model (Stark et al., 2005). It can recover individuals' absolute latent trait locations and has been allowed to use in different type of forced-choice blocks (Hontangas et al., 2015). Hence, it has been used successfully to construct new forced-choice questionnaires yielding normative measurement. Unfortunately, the MUPP model cannot solve all the problems of ipsative data in existing forced-choice questionnaires, because item parameters have been estimated from single-stimulus trials during test construction (Brown and Maydeu-Olivares, 2013). That is to say, parameters are assumed to be known in model estimation. In contrast, the Thurstonian IRT model can get model parameter estimates, based on structural equation modeling. Moreover, using the Thurstonian IRT model, there is no need to estimate items in single-stimulus trials beforehand, which is cost-saving and time efficient. But the future research could compare the performance of the MUPP response format and the mixed response format proposed here and might find that each format has its most appropriate application conditions.

### Guidance for personality test design

Based on the results of this study, some brief suggestions about the procedure and rules of the test design are offered to obtain fake-resistant personality questionnaires with high reliability, and accurate parameter recovery concomitantly.

The first step is to determine the test structure (the number of traits) and statements measuring each dimension, founded on psychological theories and other previous literature.

The second step is to assess the social desirability of each statement for the resistance to faking.

The third step is to determine which type of tests should be used. It is recommended that the test format combining the Likert scale and the forced-choice questionnaire should be used if the test only measures a small number of traits. When the number of traits is large, researchers could choose either the integrated test or the forced-choice test.

The fourth step is to construct the test. If researchers choose the combined test format, they should be quite cautious to choose items as Likert items to avoid response biases as much as possible. To resist faking, researchers should first consider items with lowest social desirability in each dimension. Then they should also use other approaches of diminishing other response biases to construct Likert items according to the situation. In most conditions, 20% of Likert items are sufficient for the test. The remaining items are used to design the forced-choice blocks, i.e., two items from different traits are matched on the social desirability to form pairs, and it's better to include some blocks composed of items keyed in opposite directions in the test. Alternatively, if the traditional forced-choice test is chosen, researchers should refer to the rules of test construction in Brown and Maydeu-Olivares (2011).

## Author contributions

YX contributes the most to the article. HYL is the corresponding author who organizes and helps conducting the analysis. HL helps provide useful suggestions on modeling and revising the article.

## Funding

This article is supported by National Natural Science Foundation of China (31571152), the Fundamental Research Funds for the Central Universities, Beijing Advanced Innovation Center for Future Education, and Special Found for Beijing Common Construction Project (019-105812).

### Conflict of interest statement

The authors declare that the research was conducted in the absence of any commercial or financial relationships that could be construed as a potential conflict of interest.
