# Supplementary material for: Integration of the Forced-Choice Questionnaire and the Likert Scale: A Simulation Study
Source: Front Psychol. 2017 May 18;8:806. doi: 10.3389/fpsyg.2017.00806 (PMC5435816; doi:10.3389/fpsyg.2017.00806)
Supplement: Supplementary file 1 [file Table1.DOCX]

Supplementary Material

Extension of the Thurstonian IRT Model: Integration of the Forced-choice Questionnaire and the Likert Scale

**Yue Xiao^1^, Hongyun Liu1^*^, Hui Li^1^**

*** Correspondence:** Hongyun Liu: hyliu@bnu.edu.cn

# Supplementary Table

Table 1. RMSE Values for Parameter Estimates and Standard Errors in 32 Conditions

| Number  of  traits | Test length^a^ | Percentage  of Likert items | Proportion of pairs composed of items keyed in opposite directions | Thresholds of  forced-choice item | | Loadings of  Likert items | | Loadings of  forced-choice items | | Intertrait  correlations | |
| --- | --- | --- | --- | --- | --- | --- | --- | --- | --- | --- | --- |
|  |  |  |  | Estimate | *SE* | Estimate | *SE* | Estimate | *SE* | Estimate | *SE* |
| 2 | 5:1 | 0% | 0% | 0.068 | 0.091 |  |  | 0.270 | 0.281 | 0.168 | 0.103 |
| 2 | 5:1 | 20% | 0% | 0.048 | 0.004 | 0.272 | 0.667 | 0.111 | 0.022 | 0.069 | 0.006 |
| 2 | 5:1 | 40% | 0% | 0.053 | 0.004 | 0.061 | 0.007 | 0.075 | 0.007 | 0.042 | 0.002 |
| 2 | 5:1 | 60% | 0% | 0.051 | 0.004 | 0.048 | 0.004 | 0.071 | 0.006 | 0.036 | 0.004 |
| 2 | 5:1 | 0% | 20% | 0.070 | 0.082 |  |  | 0.182 | 0.257 | 0.116 | 0.017 |
| 2 | 5:1 | 20% | 20% | 0.055 | 0.004 | 0.051 | 0.007 | 0.087 | 0.009 | 0.065 | 0.005 |
| 2 | 5:1 | 40% | 20% | 0.054 | 0.006 | 0.046 | 0.003 | 0.080 | 0.010 | 0.050 | 0.002 |
| 2 | 5:1 | 60% | 20% | 0.053 | 0.004 | 0.048 | 0.004 | 0.075 | 0.006 | 0.033 | 0.004 |
| 2 | 10:1 | 0% | 0% | 0.059 | 0.019 |  |  | 0.198 | 0.090 | 0.119 | 0.029 |
| 2 | 10:1 | 20% | 0% | 0.051 | 0.004 | 0.054 | 0.006 | 0.072 | 0.005 | 0.045 | 0.004 |
| 2 | 10:1 | 40% | 0% | 0.052 | 0.004 | 0.040 | 0.004 | 0.063 | 0.005 | 0.033 | 0.001 |
| 2 | 10:1 | 60% | 0% | 0.052 | 0.004 | 0.038 | 0.002 | 0.062 | 0.006 | 0.024 | 0.005 |
| 2 | 10:1 | 0% | 20% | 0.113 | 0.027 |  |  | 0.126 | 0.049 | 0.126 | 0.015 |
| 2 | 10:1 | 20% | 20% | 0.051 | 0.003 | 0.043 | 0.002 | 0.066 | 0.004 | 0.037 | 0.003 |
| 2 | 10:1 | 40% | 20% | 0.053 | 0.003 | 0.039 | 0.003 | 0.062 | 0.005 | 0.032 | 0.001 |
| 2 | 10:1 | 60% | 20% | 0.052 | 0.004 | 0.038 | 0.002 | 0.062 | 0.005 | 0.024 | 0.005 |
| 5 | 5:1 | 0% | 0% | 0.053 | 0.004 |  |  | 0.089 | 0.010 | 0.050 | 0.003 |
| 5 | 5:1 | 20% | 0% | 0.051 | 0.003 | 0.058 | 0.007 | 0.080 | 0.007 | 0.046 | 0.004 |
| 5 | 5:1 | 40% | 0% | 0.053 | 0.004 | 0.050 | 0.005 | 0.077 | 0.007 | 0.037 | 0.002 |
| 5 | 5:1 | 60% | 0% | 0.053 | 0.004 | 0.044 | 0.005 | 0.076 | 0.007 | 0.035 | 0.002 |
| 5 | 5:1 | 0% | 20% | 0.052 | 0.004 |  |  | 0.088 | 0.009 | 0.056 | 0.005 |
| 5 | 5:1 | 20% | 20% | 0.055 | 0.004 | 0.051 | 0.005 | 0.084 | 0.009 | 0.042 | 0.003 |
| 5 | 5:1 | 40% | 20% | 0.053 | 0.004 | 0.049 | 0.004 | 0.079 | 0.007 | 0.036 | 0.002 |
| 5 | 5:1 | 60% | 20% | 0.055 | 0.005 | 0.048 | 0.004 | 0.078 | 0.007 | 0.034 | 0.002 |
| 5 | 10:1 | 0% | 0% | 0.054 | 0.004 |  |  | 0.075 | 0.006 | 0.048 | 0.003 |
| 5 | 10:1 | 20% | 0% | 0.051 | 0.003 | 0.041 | 0.004 | 0.068 | 0.005 | 0.035 | 0.002 |
| 5 | 10:1 | 40% | 0% | 0.051 | 0.003 | 0.038 | 0.002 | 0.064 | 0.005 | 0.029 | 0.002 |
| 5 | 10:1 | 60% | 0% | 0.052 | 0.004 | 0.037 | 0.003 | 0.062 | 0.004 | 0.028 | 0.002 |
| 5 | 10:1 | 0% | 20% | 0.052 | 0.004 |  |  | 0.069 | 0.006 | 0.034 | 0.002 |
| 5 | 10:1 | 20% | 20% | 0.053 | 0.004 | 0.041 | 0.004 | 0.066 | 0.005 | 0.031 | 0.003 |
| 5 | 10:1 | 40% | 20% | 0.053 | 0.003 | 0.037 | 0.003 | 0.067 | 0.005 | 0.030 | 0.002 |
| 5 | 10:1 | 60% | 20% | 0.052 | 0.004 | 0.039 | 0.003 | 0.064 | 0.005 | 0.028 | 0.002 |

^a^ The test length is expressed as the ratio of the number of all questions in the test to the number of traits.
